# 1-(3-Phenyl­isoxazol-5-yl)cyclo­hexane-1,2-diol

**DOI:** 10.1107/S1600536809020947

**Published:** 2009-06-06

**Authors:** Luis Astudillo, Iván Brito, Gabriel Vallejos, Margarita Gutíerrez, Matías López-Rodríguez

**Affiliations:** a Laboratorio de Síntesis Orgánica, Instituto de Química de Recursos Naturales, Universidad de Talca, Casilla 747, Talca, Chile; b Departamento de Química, Facultad de Ciencias Básicas, Universidad de Antofagasta, Casilla 170, Antofagasta, Chile; c Laboratorio de Bioorgánica, Instituto de Química, Facultad de Ciencias, Universidad Austral de Chile, Casilla 567, Isla Teja S/N, Valdivia, Chile; d Instituto de Bio-Orgánica ‘Antonio González’, Universidad de La Laguna, Astrofísico Francisco Sánchez N°2, La Laguna, Tenerife, Spain

## Abstract

In the title compound, C_15_H_17_NO_3_, there are two mol­ecules in the asymmetric unit wherein the isoxazole rings make dihedral angles of 16.16 (15) and 16.79 (13)° with the benzene rings, and the cyclo­hexane rings adopt chair conformations. In both mol­ecules, the hydroxyl groups of the diol fragments are *cis* oriented, the O—C—C—O torsion angles being 60.76 (12) and −55.86 (11)°. The two mol­ecules are linked by a strong O—H⋯N hydrogen bond and the crystal packing is stabilized by one O—H⋯N and two O—H⋯O hydrogen bonds. An intra­molecular O—H⋯O hydrogen bond is observed in one of the mol­ecules.

## Related literature

For the uses of potassium permanganate in functional group inter­conversion inorganic chemistry, see: Singh & Lee (2001 ▶). For the use of permanganate in the preparation of natural products, see: Brown *et al.* (2008 ▶); Morris *et al.* (2009 ▶). For isoxazoles as versatile building blocks in organic synthesis, see: Melo (2005 ▶). For the synthesis, see: Hansen *et al.* (2005 ▶). For a related structure, see: Vallejos *et al.* (2009 ▶). For puckering parameters, see: Cremer & Pople (1975 ▶).
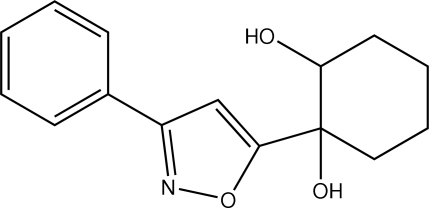


## Experimental

### 

#### Crystal data

C_15_H_17_NO_3_
*M* *_r_* = 259.3
Triclinic, 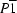

*a* = 9.4894 (17) Å
*b* = 11.5593 (15) Å
*c* = 14.0083 (13) Å
α = 73.02 (2)°
β = 81.62 (4)°
γ = 66.71 (5)°
*V* = 1349.0 (3) Å^3^
*Z* = 4
Mo *K*α radiation
μ = 0.09 mm^−1^
*T* = 298 K
0.21 × 0.10 × 0.09 mm

#### Data collection

Nonius KappaCCD area-detector diffractometer
Absorption correction: none
10826 measured reflections
5863 independent reflections
4447 reflections with *I* > 2σ(*I*)
*R*_int_ = 0.074

#### Refinement

*R*[*F* ^2^ > 2σ(*F* ^2^)] = 0.063
*wR*(*F* ^2^) = 0.168
*S* = 1.14
5863 reflections
347 parameters
H-atom parameters constrained
Δρ_max_ = 0.24 e Å^−3^
Δρ_min_ = −0.19 e Å^−3^

### 

Data collection: *COLLECT* (Nonius, 2000 ▶); cell refinement: *DENZO-SMN* (Otwinowski & Minor, 1997 ▶); data reduction: *DENZO-SMN*; program(s) used to solve structure: *SIR97* (Altomare *et al.*, 1999 ▶); program(s) used to refine structure: *SHELXL97* (Sheldrick, 2008 ▶); molecular graphics: *ORTEP-3 for Windows* (Farrugia, 1997 ▶) and *PLATON* (Spek, 2009 ▶); software used to prepare material for publication: *WinGX* (Farrugia, 1999 ▶).

## Supplementary Material

Crystal structure: contains datablocks global, I. DOI: 10.1107/S1600536809020947/pv2162sup1.cif

Structure factors: contains datablocks I. DOI: 10.1107/S1600536809020947/pv2162Isup2.hkl

Additional supplementary materials:  crystallographic information; 3D view; checkCIF report

## Figures and Tables

**Table 1:** Hydrogen-bond geometry (Å, °)

*D*—H⋯*A*	*D*—H	H⋯*A*	*D*⋯*A*	*D*—H⋯*A*
O2—H2⋯O3	0.82	2.42	2.822 (2)	111
O2—H2⋯N2^i^	0.82	2.32	3.070 (3)	153
O3—H3⋯O5^ii^	0.82	2.27	3.054 (2)	159
O5—H5⋯O3^iii^	0.82	2.12	2.923 (3)	167
O6—H6⋯N1	0.82	2.10	2.915 (3)	173

Symmetry codes: (i) 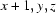; (ii) 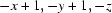; (iii) 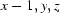.

**Fig. 1.**
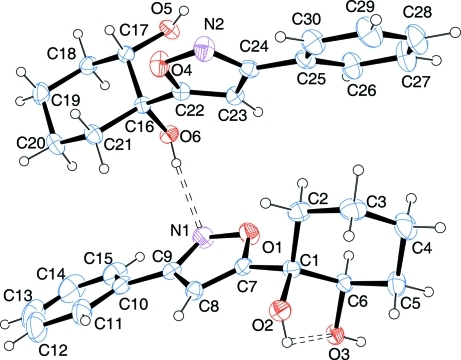
An ORTEP-3 (Farrugia, 1997) view of the two molecules in the asymmetric unit of the title compound with the atomic numbering scheme. Displacement ellipsoids are drawn at the 30% probability level. H atoms are drawn as small spheres of arbitrary radii and intramolecular hydrogen bonds indicated by dashed lines.

**Fig. 2.**
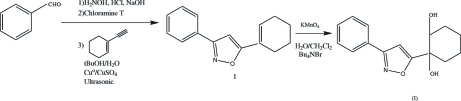
The formation of the title compound.

## supplementary crystallographic information

### Comment

Potassium permanganate has been used vastly in functional group interconversion inorganic chemistry, (*e.g.,* to oxidize alcohols to carbonyl compounds, for the cleavage or oxidation of carbon-carbon double bonds, oxidation of diols to lactones, and sulfides to sulfones (Singh & Lee, 2001). Permanganate has been used for the preparation of natural products as sylvaticin (Brown *et al.*, 2008) and membrarollin (Morris *et al.*, 2009) among others. In our search for bioactive nitrogen-containing compounds, we decided to oxidize 5-cyclohex-1-enyl-3-phenylisoxazole utilizing permanganate to obtain the title compound, since isoxazoles are a class of heterocyclic compounds having a remarkable number of applications and have been demonstrated to be very versatile building blocks in organic synthesis (Melo, 2005). We report here the crystal structure of the title compound.

In the crystal structure of the title compound, there two molecules in the asymmetric unit (Fig. 1). The isoxazole rings in the two molecules make dihedral angles of 16.16 (15) and 16.79 (12)° with the phenyl rings. In both molecules, the hydroxyl groups of the diol fragments are *cis* oriented, the O—C—C—O torsion angles being 60.76 (12) and -55.86 (11)°. The cyclohexane rings in both molecules adopt chair conformations as shown by the Cremer & Pople (1975) puckering parameters: *Q* = 0.570 (2) and 0.571 (3) Å, θ = 1.0 (2) and 0.0 (3)°, and φ = 292 (19) and 279 (9)°], respectively. Both of the molecules are linked by a strong O—H···N hydrogen bond and the crystal packing is stabilized by one O—H···N and two O—H···O type hydrogen bonds. Intramolecular O—H···O hydrogen bonds are observed in one of the molecules; details of hydrogen bonding geometry have been provided in Table 1. The crystal structure of a very closely related compound to (I) has been recently reported from our laboratory (Vallejos *et al.*, 2009).

### Experimental

*5-Cyclohex-1-enyl-3-phenylisoxazole*1 was prepared according to the procedure described by Hansen *et al.* (2005), from benzaldehyde (2.00 ml, 20 mmol, Merck), hydroxylamine hydrochloride (1.46 g, 21 mmol), chloramine-T trihydrate (5.9 g, 21 mmol) and 1-ethynylcyclohexene (2.25 ml, 21 mmol, Aldrich), giving off-white solid (yield 93%).

1-(3-Phenyl-5-isoxazolyl)-1,2-cyclohexanediol (II), Scheme 2: To a mixture of dichloromethane (25 ml), water (25 ml) and tetrabutylammonium bromide (1.00 g) as phase transfer catalyst, were added 5-cyclohex-1-enyl-3-phenylisoxazole 1 (1.00 g, 4.4 mmol) and KMnO_4_ (2.78 g, 17.6 mmol). After dissolution, the reaction mixture was cooled at 273–283 K and vigorously stirred. Thereafter, the reaction mixture was sonicated for 30 min. The reaction was then quenched with sufficient ice cooled saturated Na_2_S_2_O_5_ (aq)to dissolve all of the manganese salts and the aqueous layer was saturated with NaCl then extracted repeatedly using dichloromethane. The organic layer was separated and dried (Na_2_SO_4_), concentrated *in vacuo* and the resulting product was purified by column chromatography (silica gel, petroleum ether, EtOAc) to afford pure title compound, giving off-white solid (yield 32%). Yellow block-shaped crystals of the title compound suitable for X-ray analysis were grown from a hexane/EtOAc solution (1:1 *v*/*v*) at 298 K over a period of a few days.

### Refinement

C-bound H atoms were positioned geometrically with C—H = 0.93–0.98 Å and refined as riding model, with *U*_iso_(H) = 1.2 *U*_eq_(C) (for CH and CH_2_) or 1.5 times *U*_eq_(C) (for CH_3_). O—H distances was constrained to 0.82 Å; *U*_iso_(H) values were set at 1.2 *U*_eq_(O) of the attached atom.

### Crystal data

**Table:**

C_15_H_17_NO_3_	*Z* = 4
*M**_r_* = 259.3	*F*(000) = 552
Triclinic, *P*1	*D*_x_ = 1.277 Mg m^−^^3^
Hall symbol: -P 1	Mo *K*α radiation, λ = 0.71069 Å
*a* = 9.4894 (17) Å	Cell parameters from 5863 reflections
*b* = 11.5593 (15) Å	θ = 1.9–27.1°
*c* = 14.0083 (13) Å	µ = 0.09 mm^−^^1^
α = 73.02 (2)°	*T* = 298 K
β = 81.62 (4)°	Block, yellow
γ = 66.71 (5)°	0.21 × 0.10 × 0.09 mm
*V* = 1349.0 (3) Å^3^	

### Data collection

**Table:**

Nonius KappaCCD area-detector diffractometer	4447 reflections with *I* > 2σ(*I*)
Radiation source: fine-focus sealed tube	*R*_int_ = 0.074
graphite	θ_max_ = 27.1°, θ_min_ = 2.0°
φ scans, and ω scans with κ offsets	*h* = 0→12
10826 measured reflections	*k* = −13→14
5863 independent reflections	*l* = −17→17

### Refinement

**Table:**

Refinement on *F*^2^	Primary atom site location: structure-invariant direct methods
Least-squares matrix: full	Secondary atom site location: difference Fourier map
*R*[*F*^2^ > 2σ(*F*^2^)] = 0.063	Hydrogen site location: inferred from neighbouring sites
*wR*(*F*^2^) = 0.168	H-atom parameters constrained
*S* = 1.14	*w* = 1/[σ^2^(*F*_o_^2^) + (0.0709*P*)^2^ + 0.2904*P*] where *P* = (*F*_o_^2^ + 2*F*_c_^2^)/3
5863 reflections	(Δ/σ)_max_ < 0.001
347 parameters	Δρ_max_ = 0.24 e Å^−^^3^
0 restraints	Δρ_min_ = −0.19 e Å^−^^3^

### Special details

**Table:**

Experimental. Melting points were recorded on an Electrothermal 9100 instrument and are uncorrected; IR spectra were obtained on a Nicolet Nexus 470-FTIR spectrometer as potassium bromide pellets and are reported in wavenumbers (cm^-1^). ^1^H and ^13^CNMR spectra were measured on a Bruker AM-400 spectrometer (400 MHz), using CDCl_3 _as solvent. TMS was used as an internal standard. Chemical shifts (*d*) and *J *values are reported in p.p.m. and Hz, respectively. Reaction progress was monitored by means of thin-layer chromatography using Merck Kieselgel 60 (230–240 mesh). All reagents were purchased from Merck, Sigma and Aldrich Chemical Co. and used without further purification. Solvents were dried and distilled prior to use.*5-Cyclohex-1-enyl-3-phenylisoxazole*1: mp 361 (2) K. RMN-^1^H (CDCl_3_, 400 MHz, δ): 7,82 (2*H*, dd, J: 8,0 and 4,0); 7,45 (1*H*, m); 7,45 (2*H*, m); 6,67 (1*H*, br.s); 6,39 (1*H*, s); 2,40 (2*H*, m); 2,28 (2*H*, m); 1,80 (2*H*, m); 1,70 (2*H*, m). RMN-^13^C (CDCl3, 100 MHz, δ): 171.65, 162.44, 130.22, 129.79, 129.49, 128.85, 128.85, 126.77, 126.77, 125.41, 96.18, 25.44, 25.24, 22.12, 21.74.The title compound (II): mp 418 (2) K. RMN-^1^H (CDCl_3_, 400 MHz, δ): 7,93 (2*H*, m); 7,59 (3*H*, m); 6,82 (1*H*, s); 4,13 (1*H*, dd, *J *= 12 and 4); 1,97 (8*H*, m). RMN-^13^C (CDCl_3_,100 MHz, δ): 177.83, 161.86, 129.48, 128.42, 128.35, 128.35, 126.17, 126.17, 99.13, 73.28, 71.94, 35.11, 28.96, 23.26, 19.60. FT–IR (KBr pellet, cm^-1^): ν 3395, 2940, 2863, 1598, 1468, 1404.
Geometry. All s.u.'s (except the s.u. in the dihedral angle between two l.s. planes) are estimated using the full covariance matrix. The cell s.u.'s are taken into account individually in the estimation of s.u.'s in distances, angles and torsion angles; correlations between s.u.'s in cell parameters are only used when they are defined by crystal symmetry. An approximate (isotropic) treatment of cell s.u.'s is used for estimating s.u.'s involving l.s. planes.
Refinement. Refinement of *F*^2^ against ALL reflections. The weighted *R*-factor *wR* and goodness of fit *S* are based on *F*^2^, conventional *R*-factors *R* are based on *F*, with *F* set to zero for negative *F*^2^. The threshold expression of *F*^2^ > 2σ(*F*^2^) is used only for calculating *R*-factors(gt) *etc*. and is not relevant to the choice of reflections for refinement. *R*-factors based on *F*^2^ are statistically about twice as large as those based on *F*, and *R*- factors based on ALL data will be even larger.

### Fractional atomic coordinates and isotropic or equivalent isotropic displacement parameters (Å^2^)

**Table:**

	*x*	*y*	*z*	*U*_iso_*/*U*_eq_	
O1	0.62628 (16)	0.34815 (14)	−0.02283 (11)	0.0506 (4)	
O2	0.93980 (15)	0.08165 (12)	0.11711 (11)	0.0402 (4)	
H2	1.0099	0.109	0.1025	0.06*	
O3	0.91802 (15)	0.33990 (12)	0.08949 (10)	0.0389 (4)	
H3	0.9214	0.4003	0.1072	0.058*	
O4	0.26135 (18)	0.12795 (13)	−0.00370 (11)	0.0484 (4)	
O5	−0.00749 (16)	0.44685 (13)	−0.11911 (10)	0.0426 (4)	
H5	−0.0304	0.4079	−0.064	0.064*	
O6	0.29277 (15)	0.42716 (11)	−0.16284 (10)	0.0360 (3)	
H6	0.3837	0.4082	−0.1543	0.054*	
N1	0.6074 (2)	0.38177 (19)	−0.12650 (14)	0.0529 (5)	
N2	0.2639 (2)	0.08580 (17)	0.10155 (13)	0.0482 (5)	
C1	0.7962 (2)	0.18899 (17)	0.10844 (14)	0.0323 (4)	
C2	0.6772 (3)	0.1367 (2)	0.17032 (16)	0.0455 (5)	
H2A	0.576	0.2048	0.1596	0.055*	
H2B	0.6781	0.0648	0.1476	0.055*	
C3	0.7075 (3)	0.0904 (2)	0.28125 (18)	0.0549 (6)	
H3A	0.6256	0.0639	0.3177	0.066*	
H3B	0.803	0.0152	0.2933	0.066*	
C4	0.7175 (3)	0.1971 (3)	0.31908 (18)	0.0616 (7)	
H4A	0.7444	0.1631	0.3887	0.074*	
H4B	0.6182	0.2681	0.3145	0.074*	
C5	0.8374 (3)	0.2482 (2)	0.25845 (16)	0.0486 (6)	
H5A	0.8385	0.3189	0.2819	0.058*	
H5B	0.9382	0.1791	0.268	0.058*	
C6	0.8032 (2)	0.29647 (17)	0.14842 (14)	0.0334 (4)	
H6A	0.7037	0.3694	0.1392	0.04*	
C7	0.7557 (2)	0.24037 (17)	0.00041 (15)	0.0331 (4)	
C8	0.8209 (2)	0.20197 (17)	−0.08249 (14)	0.0346 (4)	
H8	0.9095	0.1303	−0.0875	0.042*	
C9	0.7250 (2)	0.29443 (19)	−0.16047 (15)	0.0378 (5)	
C10	0.7449 (2)	0.3044 (2)	−0.26858 (16)	0.0437 (5)	
C11	0.8477 (3)	0.2006 (3)	−0.30373 (18)	0.0608 (7)	
H11	0.9012	0.1227	−0.2588	0.073*	
C12	0.8715 (4)	0.2119 (4)	−0.4055 (2)	0.0849 (9)	
H12	0.9409	0.1421	−0.429	0.102*	
C13	0.7913 (5)	0.3277 (4)	−0.4720 (2)	0.0929 (11)	
H13	0.8073	0.3359	−0.5403	0.111*	
C14	0.6878 (4)	0.4311 (4)	−0.4375 (2)	0.0854 (10)	
H14	0.6336	0.5085	−0.4826	0.102*	
C15	0.6643 (3)	0.4202 (3)	−0.3370 (2)	0.0629 (7)	
H15	0.5942	0.4904	−0.3142	0.075*	
C16	0.2624 (2)	0.31090 (16)	−0.14292 (14)	0.0303 (4)	
C17	0.0942 (2)	0.35473 (18)	−0.17172 (14)	0.0343 (4)	
H17	0.0657	0.2781	−0.1537	0.041*	
C18	0.0737 (2)	0.4167 (2)	−0.28265 (15)	0.0412 (5)	
H18A	0.0924	0.4972	−0.3002	0.049*	
H18B	−0.0314	0.438	−0.2984	0.049*	
C19	0.1827 (3)	0.3262 (2)	−0.34394 (17)	0.0520 (6)	
H19A	0.1708	0.3705	−0.4144	0.062*	
H19B	0.1567	0.2498	−0.3318	0.062*	
C20	0.3488 (3)	0.2838 (2)	−0.31744 (16)	0.0493 (6)	
H20A	0.378	0.3592	−0.336	0.059*	
H20B	0.415	0.2227	−0.355	0.059*	
C21	0.3705 (2)	0.22007 (19)	−0.20610 (15)	0.0407 (5)	
H21A	0.4761	0.1977	−0.1908	0.049*	
H21B	0.3508	0.1401	−0.1889	0.049*	
C22	0.2762 (2)	0.24539 (17)	−0.03319 (14)	0.0329 (4)	
C23	0.2892 (2)	0.28088 (18)	0.04736 (14)	0.0347 (4)	
H23	0.301	0.3566	0.0485	0.042*	
C24	0.2811 (2)	0.17773 (18)	0.13034 (15)	0.0353 (4)	
C25	0.2852 (2)	0.16502 (19)	0.23812 (15)	0.0379 (5)	
C26	0.2603 (3)	0.2727 (2)	0.27237 (17)	0.0526 (6)	
H26	0.2419	0.3537	0.2273	0.063*	
C27	0.2626 (3)	0.2598 (3)	0.37386 (18)	0.0667 (7)	
H27	0.2458	0.3324	0.3965	0.08*	
C28	0.2896 (3)	0.1406 (3)	0.44105 (18)	0.0661 (7)	
H28	0.2912	0.1325	0.509	0.079*	
C29	0.3143 (3)	0.0333 (3)	0.40761 (18)	0.0659 (7)	
H29	0.3331	−0.0476	0.453	0.079*	
C30	0.3113 (3)	0.0452 (2)	0.30692 (17)	0.0509 (6)	
H30	0.327	−0.0277	0.285	0.061*	

### Atomic displacement parameters (Å^2^)

**Table:**

	*U*^11^	*U*^22^	*U*^33^	*U*^12^	*U*^13^	*U*^23^
O1	0.0387 (8)	0.0564 (9)	0.0423 (10)	0.0013 (7)	−0.0070 (7)	−0.0159 (7)
O2	0.0374 (8)	0.0297 (7)	0.0493 (9)	−0.0067 (6)	−0.0041 (7)	−0.0111 (6)
O3	0.0488 (8)	0.0377 (7)	0.0382 (8)	−0.0244 (7)	0.0061 (6)	−0.0136 (6)
O4	0.0746 (11)	0.0373 (8)	0.0361 (9)	−0.0270 (7)	−0.0048 (7)	−0.0037 (6)
O5	0.0376 (8)	0.0449 (8)	0.0347 (9)	−0.0076 (6)	0.0039 (6)	−0.0083 (6)
O6	0.0363 (7)	0.0292 (7)	0.0428 (9)	−0.0127 (6)	−0.0061 (6)	−0.0066 (6)
N1	0.0414 (11)	0.0634 (12)	0.0424 (12)	−0.0062 (9)	−0.0125 (9)	−0.0105 (9)
N2	0.0678 (13)	0.0428 (10)	0.0324 (10)	−0.0244 (9)	−0.0056 (9)	−0.0002 (8)
C1	0.0316 (10)	0.0282 (9)	0.0366 (11)	−0.0097 (8)	−0.0005 (8)	−0.0096 (8)
C2	0.0449 (12)	0.0450 (11)	0.0502 (14)	−0.0237 (10)	−0.0007 (10)	−0.0080 (10)
C3	0.0565 (14)	0.0565 (13)	0.0504 (15)	−0.0330 (12)	0.0049 (11)	0.0019 (11)
C4	0.0812 (18)	0.0710 (16)	0.0356 (14)	−0.0389 (14)	0.0104 (12)	−0.0091 (12)
C5	0.0709 (15)	0.0504 (12)	0.0323 (12)	−0.0313 (12)	−0.0010 (10)	−0.0102 (10)
C6	0.0379 (11)	0.0331 (9)	0.0303 (11)	−0.0153 (8)	0.0044 (8)	−0.0095 (8)
C7	0.0317 (10)	0.0306 (9)	0.0384 (12)	−0.0113 (8)	−0.0046 (8)	−0.0099 (8)
C8	0.0367 (11)	0.0319 (9)	0.0360 (12)	−0.0120 (8)	−0.0031 (9)	−0.0100 (8)
C9	0.0345 (11)	0.0415 (11)	0.0413 (12)	−0.0184 (9)	−0.0049 (9)	−0.0090 (9)
C10	0.0466 (12)	0.0580 (13)	0.0354 (12)	−0.0322 (11)	−0.0065 (10)	−0.0048 (10)
C11	0.0778 (18)	0.0710 (16)	0.0418 (15)	−0.0347 (14)	−0.0020 (12)	−0.0168 (12)
C12	0.116 (3)	0.112 (3)	0.0461 (18)	−0.058 (2)	0.0105 (17)	−0.0336 (18)
C13	0.138 (3)	0.140 (3)	0.0335 (17)	−0.094 (3)	−0.0026 (19)	−0.012 (2)
C14	0.111 (3)	0.106 (2)	0.0452 (18)	−0.064 (2)	−0.0234 (17)	0.0146 (17)
C15	0.0673 (17)	0.0697 (16)	0.0512 (16)	−0.0345 (14)	−0.0148 (13)	0.0040 (13)
C16	0.0343 (10)	0.0267 (9)	0.0299 (11)	−0.0122 (8)	−0.0009 (8)	−0.0060 (8)
C17	0.0353 (10)	0.0364 (10)	0.0326 (11)	−0.0153 (8)	0.0002 (8)	−0.0086 (8)
C18	0.0408 (12)	0.0500 (12)	0.0346 (12)	−0.0195 (10)	−0.0085 (9)	−0.0066 (9)
C19	0.0661 (16)	0.0640 (14)	0.0348 (13)	−0.0294 (12)	−0.0014 (11)	−0.0185 (11)
C20	0.0551 (14)	0.0544 (13)	0.0376 (13)	−0.0178 (11)	0.0100 (10)	−0.0198 (11)
C21	0.0422 (12)	0.0364 (10)	0.0413 (13)	−0.0113 (9)	0.0041 (9)	−0.0142 (9)
C22	0.0319 (10)	0.0275 (9)	0.0352 (11)	−0.0086 (8)	−0.0029 (8)	−0.0047 (8)
C23	0.0356 (11)	0.0332 (10)	0.0335 (11)	−0.0119 (8)	−0.0040 (8)	−0.0056 (8)
C24	0.0278 (10)	0.0364 (10)	0.0352 (12)	−0.0080 (8)	−0.0015 (8)	−0.0052 (8)
C25	0.0303 (10)	0.0426 (11)	0.0320 (12)	−0.0088 (9)	−0.0005 (8)	−0.0038 (9)
C26	0.0677 (16)	0.0437 (12)	0.0361 (13)	−0.0136 (11)	−0.0033 (11)	−0.0046 (10)
C27	0.095 (2)	0.0624 (15)	0.0375 (15)	−0.0225 (15)	−0.0020 (13)	−0.0152 (12)
C28	0.0800 (19)	0.0768 (18)	0.0289 (13)	−0.0212 (15)	−0.0005 (12)	−0.0077 (12)
C29	0.0844 (19)	0.0591 (15)	0.0358 (14)	−0.0216 (14)	0.0008 (13)	0.0058 (11)
C30	0.0589 (15)	0.0455 (12)	0.0404 (14)	−0.0171 (11)	−0.0002 (11)	−0.0037 (10)

### Geometric parameters (Å, °)

**Table:**

O1—C7	1.355 (2)	C12—C13	1.382 (5)
O1—N1	1.408 (2)	C12—H12	0.93
O2—C1	1.426 (2)	C13—C14	1.377 (5)
O2—H2	0.82	C13—H13	0.93
O3—C6	1.432 (2)	C14—C15	1.369 (4)
O3—H3	0.82	C14—H14	0.93
O4—C22	1.356 (2)	C15—H15	0.93
O4—N2	1.412 (2)	C16—C22	1.501 (3)
O5—C17	1.431 (2)	C16—C21	1.538 (3)
O5—H5	0.82	C16—C17	1.547 (3)
O6—C16	1.425 (2)	C17—C18	1.515 (3)
O6—H6	0.82	C17—H17	0.98
N1—C9	1.314 (3)	C18—C19	1.521 (3)
N2—C24	1.314 (3)	C18—H18A	0.97
C1—C7	1.501 (3)	C18—H18B	0.97
C1—C2	1.530 (3)	C19—C20	1.522 (3)
C1—C6	1.531 (2)	C19—H19A	0.97
C2—C3	1.519 (3)	C19—H19B	0.97
C2—H2A	0.97	C20—C21	1.523 (3)
C2—H2B	0.97	C20—H20A	0.97
C3—C4	1.517 (3)	C20—H20B	0.97
C3—H3A	0.97	C21—H21A	0.97
C3—H3B	0.97	C21—H21B	0.97
C4—C5	1.524 (3)	C22—C23	1.345 (3)
C4—H4A	0.97	C23—C24	1.420 (3)
C4—H4B	0.97	C23—H23	0.93
C5—C6	1.514 (3)	C24—C25	1.478 (3)
C5—H5A	0.97	C25—C26	1.385 (3)
C5—H5B	0.97	C25—C30	1.388 (3)
C6—H6A	0.98	C26—C27	1.388 (3)
C7—C8	1.343 (3)	C26—H26	0.93
C8—C9	1.415 (3)	C27—C28	1.372 (4)
C8—H8	0.93	C27—H27	0.93
C9—C10	1.475 (3)	C28—C29	1.376 (4)
C10—C11	1.383 (3)	C28—H28	0.93
C10—C15	1.397 (3)	C29—C30	1.380 (3)
C11—C12	1.386 (4)	C29—H29	0.93
C11—H11	0.93	C30—H30	0.93
			
C7—O1—N1	108.43 (15)	C13—C14—H14	119.9
C1—O2—H2	109.5	C14—C15—C10	120.4 (3)
C6—O3—H3	109.5	C14—C15—H15	119.8
C22—O4—N2	108.55 (14)	C10—C15—H15	119.8
C17—O5—H5	109.5	O6—C16—C22	109.15 (14)
C16—O6—H6	109.5	O6—C16—C21	110.93 (15)
C9—N1—O1	105.42 (17)	C22—C16—C21	111.75 (15)
C24—N2—O4	105.51 (15)	O6—C16—C17	106.02 (14)
O2—C1—C7	108.33 (15)	C22—C16—C17	109.02 (15)
O2—C1—C2	107.01 (15)	C21—C16—C17	109.80 (15)
C7—C1—C2	110.19 (16)	O5—C17—C18	108.31 (16)
O2—C1—C6	111.29 (15)	O5—C17—C16	110.79 (15)
C7—C1—C6	110.53 (15)	C18—C17—C16	111.75 (16)
C2—C1—C6	109.43 (16)	O5—C17—H17	108.6
C3—C2—C1	112.45 (17)	C18—C17—H17	108.6
C3—C2—H2A	109.1	C16—C17—H17	108.6
C1—C2—H2A	109.1	C17—C18—C19	111.44 (18)
C3—C2—H2B	109.1	C17—C18—H18A	109.3
C1—C2—H2B	109.1	C19—C18—H18A	109.3
H2A—C2—H2B	107.8	C17—C18—H18B	109.3
C4—C3—C2	111.14 (18)	C19—C18—H18B	109.3
C4—C3—H3A	109.4	H18A—C18—H18B	108
C2—C3—H3A	109.4	C18—C19—C20	111.25 (17)
C4—C3—H3B	109.4	C18—C19—H19A	109.4
C2—C3—H3B	109.4	C20—C19—H19A	109.4
H3A—C3—H3B	108	C18—C19—H19B	109.4
C3—C4—C5	110.92 (19)	C20—C19—H19B	109.4
C3—C4—H4A	109.5	H19A—C19—H19B	108
C5—C4—H4A	109.5	C19—C20—C21	111.04 (18)
C3—C4—H4B	109.5	C19—C20—H20A	109.4
C5—C4—H4B	109.5	C21—C20—H20A	109.4
H4A—C4—H4B	108	C19—C20—H20B	109.4
C6—C5—C4	110.94 (19)	C21—C20—H20B	109.4
C6—C5—H5A	109.5	H20A—C20—H20B	108
C4—C5—H5A	109.5	C20—C21—C16	111.55 (16)
C6—C5—H5B	109.5	C20—C21—H21A	109.3
C4—C5—H5B	109.5	C16—C21—H21A	109.3
H5A—C5—H5B	108	C20—C21—H21B	109.3
O3—C6—C5	111.89 (16)	C16—C21—H21B	109.3
O3—C6—C1	107.13 (14)	H21A—C21—H21B	108
C5—C6—C1	111.39 (15)	C23—C22—O4	109.57 (17)
O3—C6—H6A	108.8	C23—C22—C16	134.23 (16)
C5—C6—H6A	108.8	O4—C22—C16	115.99 (15)
C1—C6—H6A	108.8	C22—C23—C24	105.08 (16)
C8—C7—O1	109.78 (17)	C22—C23—H23	127.5
C8—C7—C1	133.69 (17)	C24—C23—H23	127.5
O1—C7—C1	116.53 (16)	N2—C24—C23	111.28 (17)
C7—C8—C9	104.88 (17)	N2—C24—C25	119.46 (17)
C7—C8—H8	127.6	C23—C24—C25	129.24 (17)
C9—C8—H8	127.6	C26—C25—C30	118.9 (2)
N1—C9—C8	111.47 (19)	C26—C25—C24	120.66 (18)
N1—C9—C10	119.76 (19)	C30—C25—C24	120.47 (18)
C8—C9—C10	128.74 (19)	C25—C26—C27	120.1 (2)
C11—C10—C15	119.1 (2)	C25—C26—H26	119.9
C11—C10—C9	120.4 (2)	C27—C26—H26	119.9
C15—C10—C9	120.5 (2)	C28—C27—C26	120.4 (2)
C10—C11—C12	120.4 (3)	C28—C27—H27	119.8
C10—C11—H11	119.8	C26—C27—H27	119.8
C12—C11—H11	119.8	C27—C28—C29	119.8 (2)
C13—C12—C11	119.6 (3)	C27—C28—H28	120.1
C13—C12—H12	120.2	C29—C28—H28	120.1
C11—C12—H12	120.2	C28—C29—C30	120.2 (2)
C14—C13—C12	120.4 (3)	C28—C29—H29	119.9
C14—C13—H13	119.8	C30—C29—H29	119.9
C12—C13—H13	119.8	C29—C30—C25	120.6 (2)
C15—C14—C13	120.2 (3)	C29—C30—H30	119.7
C15—C14—H14	119.9	C25—C30—H30	119.7
			
C7—O1—N1—C9	−0.3 (2)	C9—C10—C15—C14	177.2 (2)
C22—O4—N2—C24	−0.5 (2)	O6—C16—C17—O5	−55.87 (18)
O2—C1—C2—C3	65.7 (2)	C22—C16—C17—O5	61.51 (18)
C7—C1—C2—C3	−176.76 (16)	C21—C16—C17—O5	−175.77 (14)
C6—C1—C2—C3	−55.0 (2)	O6—C16—C17—C18	65.02 (18)
C1—C2—C3—C4	55.1 (3)	C22—C16—C17—C18	−177.60 (14)
C2—C3—C4—C5	−55.0 (3)	C21—C16—C17—C18	−54.87 (19)
C3—C4—C5—C6	56.5 (3)	O5—C17—C18—C19	177.89 (16)
C4—C5—C6—O3	−177.55 (17)	C16—C17—C18—C19	55.6 (2)
C4—C5—C6—C1	−57.7 (2)	C17—C18—C19—C20	−55.8 (2)
O2—C1—C6—O3	60.75 (19)	C18—C19—C20—C21	56.1 (2)
C7—C1—C6—O3	−59.67 (19)	C19—C20—C21—C16	−56.4 (2)
C2—C1—C6—O3	178.80 (16)	O6—C16—C21—C20	−61.6 (2)
O2—C1—C6—C5	−61.9 (2)	C22—C16—C21—C20	176.34 (15)
C7—C1—C6—C5	177.67 (16)	C17—C16—C21—C20	55.2 (2)
C2—C1—C6—C5	56.1 (2)	N2—O4—C22—C23	0.4 (2)
N1—O1—C7—C8	−0.5 (2)	N2—O4—C22—C16	−174.98 (15)
N1—O1—C7—C1	179.87 (14)	O6—C16—C22—C23	10.7 (3)
O2—C1—C7—C8	4.2 (3)	C21—C16—C22—C23	133.8 (2)
C2—C1—C7—C8	−112.5 (2)	C17—C16—C22—C23	−104.7 (2)
C6—C1—C7—C8	126.4 (2)	O6—C16—C22—O4	−175.34 (15)
O2—C1—C7—O1	−176.22 (14)	C21—C16—C22—O4	−52.3 (2)
C2—C1—C7—O1	67.0 (2)	C17—C16—C22—O4	69.3 (2)
C6—C1—C7—O1	−54.0 (2)	O4—C22—C23—C24	−0.2 (2)
O1—C7—C8—C9	1.01 (19)	C16—C22—C23—C24	174.04 (19)
C1—C7—C8—C9	−179.40 (18)	O4—N2—C24—C23	0.4 (2)
O1—N1—C9—C8	1.0 (2)	O4—N2—C24—C25	178.74 (16)
O1—N1—C9—C10	−177.26 (15)	C22—C23—C24—N2	−0.1 (2)
C7—C8—C9—N1	−1.3 (2)	C22—C23—C24—C25	−178.28 (18)
C7—C8—C9—C10	176.78 (17)	N2—C24—C25—C26	−161.9 (2)
N1—C9—C10—C11	−166.5 (2)	C23—C24—C25—C26	16.1 (3)
C8—C9—C10—C11	15.6 (3)	N2—C24—C25—C30	16.9 (3)
N1—C9—C10—C15	15.8 (3)	C23—C24—C25—C30	−165.0 (2)
C8—C9—C10—C15	−162.2 (2)	C30—C25—C26—C27	0.4 (3)
C15—C10—C11—C12	0.7 (3)	C24—C25—C26—C27	179.3 (2)
C9—C10—C11—C12	−177.1 (2)	C25—C26—C27—C28	0.0 (4)
C10—C11—C12—C13	−0.2 (4)	C26—C27—C28—C29	−0.1 (4)
C11—C12—C13—C14	−0.4 (5)	C27—C28—C29—C30	−0.3 (4)
C12—C13—C14—C15	0.5 (5)	C28—C29—C30—C25	0.7 (4)
C13—C14—C15—C10	0.0 (4)	C26—C25—C30—C29	−0.8 (3)
C11—C10—C15—C14	−0.5 (3)	C24—C25—C30—C29	−179.6 (2)

### Hydrogen-bond geometry (Å, °)

**Table:**

*D*—H···*A*	*D*—H	H···*A*	*D*···*A*	*D*—H···*A*
O2—H2···O3	0.82	2.42	2.822 (2)	111
O6—H6···N1	0.82	2.10	2.915 (3)	173
O2—H2···N2^i^	0.82	2.32	3.070 (3)	153
O3—H3···O5^ii^	0.82	2.27	3.054 (2)	159
O5—H5···O3^iii^	0.82	2.12	2.923 (3)	167

Symmetry codes: (i) *x*+1, *y*, *z*; (ii) −*x*+1, −*y*+1, −*z*; (iii) *x*−1, *y*, *z*.
